# Early substrate-based catheter ablation vs. antiarrhythmic drug therapy for ventricular tachyarrhythmias among patients with prior myocardial infarction: the MANTRA-VT randomized trial

**DOI:** 10.1093/europace/euaf236

**Published:** 2025-10-15

**Authors:** Pekka Raatikainen, Heikki Mäkynen, Juha Hartikainen, Mats Jensen Urstad, Leena Konkola, Niels C F Sandgaard, Peter Lukac, Arne Johannessen, Anders Jönsson, Peter Schuster, Carina Blomström-Lundqvist, Jussi Kuutti, Piia Lavikainen, Hannu Parikka

**Affiliations:** Department of Cardiology, Heart and Lung Center, Helsinki University Hospital and University of Helsinki, Haartmaninkatu 4, Helsinki 00290, Finland; Department of Internal Medicine, Central Finland Central Hospital, Jyväskylä, Finland; Heart Hospital Tampere University Hospital, Tampere, Finland; Heart Center, Kuopio University Hospital, Kuopio, Finland; University of Eastern Finland, Kuopio, Finland; Department of Cardiology, Karolinska University Hospital, Stockholm, Sweden; Department of Medicine, Karolinska Institute, Stockholm, Sweden; Department of Internal Medicine, Central Finland Central Hospital, Jyväskylä, Finland; Department of Cardiology, Odense University Hospital, Odense, Denmark; Deparment of Cardiology, Aarhus University Hospital, Aarhus, Denmark; Department of Cardiology, Gentofte Hospital, Copenhagen University Hospital, Gentofte, Denmark; University of Linköping, Linköping, Sweden; Department of Heart Disease, Haukeland University Hospital, Bergen, Norway; Department of Cardiology, School of Medical Sciences, Faculty of Medicine and Health, Örebro University, Örebro, Sweden; Department of Medical Science, Uppsala University Hospital, Uppsala, Sweden; Department of Cardiology, Heart and Lung Center, Helsinki University Hospital and University of Helsinki, Haartmaninkatu 4, Helsinki 00290, Finland; University of Eastern Finland, Kuopio, Finland; Department of Cardiology, Heart and Lung Center, Helsinki University Hospital and University of Helsinki, Haartmaninkatu 4, Helsinki 00290, Finland

**Keywords:** Ventricular tachyarrhythmia, Radiofrequency catheter ablation, Antiarrhythmic drug therapy, ICD therapy, Myocardial infarction, Randomized trial

## Abstract

**Aims:**

Ventricular tachyarrhythmias (VT/VF) are common among patients with prior myocardial infarction (MI). MANTRA-VT trial was designed to compare the efficacy and safety of early substrate-based radiofrequency catheter ablation (RFCA) to antiarrhythmic drug (AAD) therapy for ventricular tachyarrhythmias.

**Methods and results:**

We randomly assigned 58 AAD naïve post MI patients with implantable cardioverter defibrillator (ICD) and at least one documented VT/VF episode after the device implantation to an initial treatment strategy of substrate-based RFCA or AAD therapy. The primary endpoint was cumulative number of ventricular tachyarrhythmias (VT/VF burden) at 12 months. The secondary endpoints included all-cause mortality, hospitalization, adverse events, and VT/VF burden at 24 months. Analyses were performed on an intention-to-treat basis. The median number of VT/VF episodes at 12 months was zero in both the RFCA (range 0–3) and the AAD group (range 0–23) (*P* = 0.454), whereas the rate of appropriate ICD shocks was 7% and 30% in the RFCA and the AAD groups (*P* = 0.026), respectively. During the extended follow-up, 82% of the patients in the RFCA group and 63% in the AAD group had no ICD therapies (*P* = 0.012). There was no significant difference between the groups in total mortality (HR 1.02, 95% CI 0.20–5.11, *P* = 0.86) and hospitalization (HR 1.35, 95% CI 0.36–5.09. *P* = 0.66) at 24 months. Therapy-related adverse events occurred in 3.6% and 16.7% of the patients in the RFCA and the AAD groups (*P* = 0.10), respectively.

**Conclusion:**

Early substrate-based RFCA was associated with reduced risk of ICD therapies, but with no meaningful difference in VT/VF burden, mortality, hospitalization, and adverse events.

## Graphical Abstract

**Graphical Abstract:**
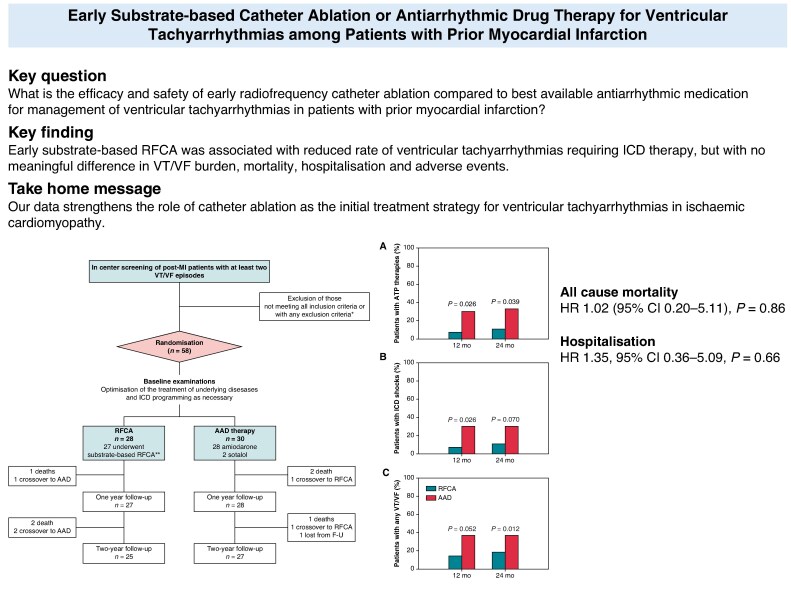

What’s new?

- MANTRA-VT trial was designed to compare the efficacy and safety of early substrate-based radiofrequency catheter ablation (RFCA) to antiarrhythmic drug (AAD) therapy for ventricular tachyarrhythmias (VT/VF).
- Early substrate-based RFCA was associated with reduced risk of implantable cardioverter defibrillator therapies, but with no meaningful difference in VT/VF burden, mortality, hospitalization, and adverse events.
- The findings strengthen the role of catheter ablation as the initial treatment strategy for ventricular tachyarrhythmias in ischaemic cardiomyopathy.

## Introduction

Implantable cardioverter defibrillator (ICD) is the cornerstone in the prevention of sudden cardiac death (SCD) among survivors of remote myocardial infarction (MI) and elevated risk of life-threatening arrhythmias.^1,2^ However, although the ICD effectively terminates ventricular tachycardia (VT) and ventricular fibrillation (VF) by antitachycardia pacing (ATP) or a shock, ICD interventions are palliative and do not cure or prevent VT/VF.

It is estimated that depending on the patient cohort, 10–50% of the ICD recipients will have adequate ICD shocks within 3 years from the device implantation.^3,4^ Traditionally, antiarrhythmic drug (AAD) therapy has been the mainstay in reducing VT/VF recurrences in ICD recipients.^5^ Amiodarone is the most potent drug available for treatment of complex atrial and ventricular arrhythmias.^6^ Among ICD recipients, it reduces the incidence of ICD shocks and ATP,^7^ but severe adverse effects and multiple drug interactions limit its long-term use.^8^ Over the last decades, radiofrequency catheter ablation (RFCA) has evolved as a promising tool for management of ventricular tachyarrhythmias among patients with prior MI.^5,9,10^ Radiofrequency catheter ablation has proven effective in the treatment of drug-refractory ventricular tachyarrhythmias^11,12^ and electric storm in patients with structural heart disease.^13–18^ In addition, there is some evidence indicating that prophylactic substrate-based catheter ablation may prevent adequate ICD therapies during long-term follow-up.^12,19^ Nevertheless, even in the light of the most recent trials,^20–25^ the optimal timing of RFCA in post-MI patients remains disputable and more information is needed to determine whether RFCA or AAD should be the first-line therapy after the first ICD shock in patient with no previous AAD therapy.

The aim of the randomized controlled Medical ANtiarrhythmic Treatment or Radiofrequency Ablation in ischemic Ventricular Tachyarrhythmias (MANTRA-VT) trial was to determine whether substrate-based RFCA is superior to amiodarone therapy in reducing VT/VF burden among antiarrhythmic drug-naïve post MI patients with at least two VT/VF episodes detected by the device or ECG. Here, we present the results for the primary and selected secondary endpoints (e.g. mortality and hospitalizations).

## Methods

### General

The MANTRA-VT trial (ClinicalTrials.gov ID NCT02303639) is an investigator-initiated, multicentre, randomized, open-labelled, prospective trial comparing the efficacy and safety of RFCA to AAD therapy in preventing VT/VF recurrencies among AAD-naïve patients with prior MI and a history of at least two VT/VF episodes documented by the device or an ECG. The last VT/VF (index) episode should be documented within the foregoing 12 months. The study was conducted in accordance with the Helsinki declaration, and the protocol was accepted by the Ethics Committee of Central Finland Hospital District and by the local ethical committees in the participating centres. All patients gave written informed consent for the study before enrolment.

After the treatment of coronary artery disease (e.g. beta-blocker, ACE inhibitor or angiotensin receptor blocker, statin, antithrombotic medication) and other underlying diseases were optimized as necessary, patients 18–80 years of age meeting all the eligibility criteria and no exclusion criteria (see Supplementary material online, *Table S1*) were randomly assigned 1:1 to early RFCA or AAD therapy in random blocks of four or eight. That is, the patients with a primary prevention indication for ICD therapy were eligible to the study after two and those with a secondary prevention indication after one appropriate ICD therapy or otherwise documented VT/VF episode after the device implantation, respectively.

### Baseline examinations and implantable cardioverter defibrillator programming

At the baseline, a full clinical evaluation including medical history, physical examination, 12-lead ECG, transthoracic echocardiography, blood samples, and ICD interrogations was performed to all study subjects. Patient-related outcome measures (PROMs) were evaluated using the following questionnaires at baseline and at 12 and 24 months: SF-36 and EQ5D to assess generic quality of life, Hospital Anxiety and Depression Scale (HADS) to assess symptoms of anxiety and depression, the Implantable Cardioverter Defibrillator Patient Concerns Questionnaire (ICDC) to assess concerns about ICD shocks, 18-item Florida Patient Acceptance Survey to assess device acceptance (FPAS), and the Minnesota Living with Heart Failure Questionnaire (MLHFQ) to assess disease-specific health status.

Implantable cardioverter defibrillator programming was standardized, and a long detection time at VT zone and a high rate for VF zone were used to avoid inadequate and unnecessary ICD therapies. The recommended detection rates were 240 b.p.m. and 170 b.p.m. (or less if a slow VT had been documented) for the VF zone and VT zone, respectively. The recommended detection time in VT zone was 30 s, while in the VF zone, the nominal detection time was used. A specific monitoring zone (140–170 b.p.m. with a detection time of 60 s) with no programmed therapies was used to detect slow VTs.

The initial therapy was ATP in the VT zone and an ICD shock with ATP during charging in the VF zone. In addition, it was recommended to use proper device programming to avoid unnecessary right ventricular pacing in patients with conventional ICDs and to ensure over 90% biventricular pacing in patients with CRT-D.

### Therapeutic interventions

The RFCA procedures were done under general anaesthesia or conscious sedation according to the institutional routine. Implantable cardioverter defibrillator tachytherapies were inactivated before the procedure. The left ventricle (LV) was accessed transseptally via left atrium, retrogradely via aorta or both ways upon the preference of the responsible electrophysiologist. The ablation catheter was manipulated manually or with remote magnetic navigation (RMN) (Stereotaxis Inc., St Louis, MO, USA). The target intraprocedural activate clotting time (ACT) was above 300 s. The maximum power during ablation was 35–50 W and the irrigation flow 15–30 mL/min.

A detailed three-dimensional (3D) electroanatomic substrate map of the LV was constructed during underlying rhythm or pacing using the Carto 3 system and an open-irrigated ablation catheter or a multipole mapping catheter (Biosense Webster, Irvine, CA, USA). Epicardial and/or right ventricular mapping and ablation were performed if clinically required. Areas with bipolar signal amplitude less than 0.5 mV were considered abnormal and those with voltage > 1.5 mV healthy. The goal of the RFCA was to eliminate fractionated signals and late potentials in the low-voltage area and to abolish inducibility of clinical and non-clinical VTs. Activation and/or pace mapping and specific pacing manoeuvres could be used to reinforce substrate mapping if needed on clinical basis. Programmed electrophysiologic stimulation was performed using at least one basic cycle length (500 ms) and with at least two extra stimuli (shortest coupling interval 200 ms) to evaluate the inducibility of the VT/VF after the ablation. In patients with VT/VF recurrence after the initial RFCA, re-ablation was encouraged, and if it was considered impractical, AAD therapy could be started (crossover).

Amiodarone was the first-line AAD. The recommended loading and maintenance doses of amiodarone were 600–1200 mg per day and 200–400 mg QD, respectively. In patients with a contraindication to amiodarone (e.g. severe pulmonary or thyroid disease), sotalol could be prescribed at the discretion of the responsible investigator. The use of class IA and IC AADs was contraindicated, but otherwise, a shift from the initial AAD to another AAD or use of a combination of multiple AADs was allowed if needed. Before crossover to catheter ablation, it was recommended to ensure that the dose of the AAD was sufficient and that a steady-state concentration had been achieved.

### Follow-up

The patients were reviewed in the institutional outpatient clinic at 3, 12, and 24 months (secondary endpoint). In addition, at 6 and 18 months, the ICD data were collected either at the outpatient clinic or via a remote monitoring system. Data on the primary outcomes and other arrhythmia episodes (e.g. atrial fibrillation), sick leave periods, contacts to healthcare units, and hospitalizations were collect at every follow-up visit. Patient-related outcome measures and echocardiography were reassessed at 12 and 24 months. The follow-up scheme is summarized in Supplementary material online, *Table S2*.

### Endpoints

The primary endpoint was VT/VF burden defined as cumulative number of all device-detected and otherwise (12 lead ECG, Holter, telemetry, etc.) documented sustained ventricular tachyarrhythmias lasting 30 s or longer during the 12 months follow-up period. No blanking period was used. The secondary endpoints are summarized in the Supplementary material online, *Table S3*. The study was not powered for all secondary objectives.

An adverse event (AE) was defined as any undesirable experience (sign, symptom, illness, abnormal laboratory value, or other medical event) occurring in a subject during the study, whether or not related to the treatment. To ensure standardized evaluation across investigational sites, assessment of seriousness, outcome, and causality of the AEs were made according to definitions and classifications presented in the Supplementary material online, *Table S4*. The serious AEs and outcomes reported by the investigators were evaluated by a clinical endpoint adjudication committee consisting of two independent medical experts, and the ascertainment of the other secondary endpoints was carried out locally.

### Statistical analysis

The reduction of the VT/VF episodes by RFCA compared the AAD therapy was expected to be 25–50%. The power calculations were made for both the high and low estimate (see Supplementary material online, *Table S5*). Based on the most like scenario, the plan was to enrol 100 patients (50 patients per group). However, due to slower than anticipated recruitment, it was decided to stop enrolment after 60 patients were included or no later than at the end of 2021 and to report the extended 24-month results at the same time as the 12-month results.

The statistical analysis was performed using SPSS 29.0 software (SPSS Inc., Chicago, IL). The date of the randomization was used as time zero for analysis, and treatment groups were compared on an intention-to-treat basis. The data are reported as means and standard deviations for normally distributed variables, and medians and interquartile ranges for continuous variables with skewed distributions and frequencies for categorical variables. An independent samples *t*-test or the Wilcoxon rank-sum test was used to analyse differences between continuous variables, and the χ² test or Fisher's exact test (when appropriate) for categorical values. A two-sided *P*-value of less than 0.05 was considered statistically significant.

## Results

### Characteristics of the patients

**Figure 1:**
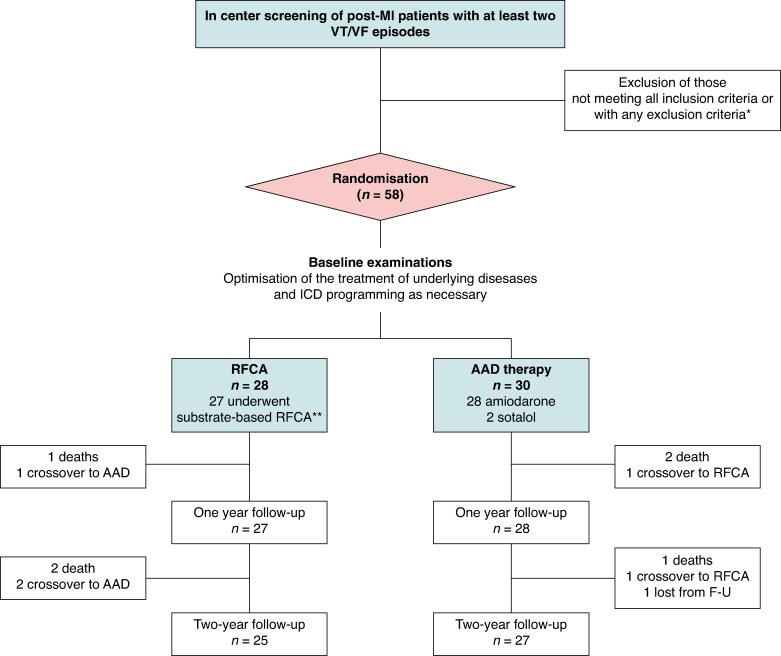
Flowchart of the MANTRA-VT trial. A total of 58 patients with at least two VT or VF episodes detected by ECG or the device were randomized to substrate-based RFCA (*n* = 28) and AAD therapy (*n* = 30). Unfortunately, the screening information was not collected systematically and no data on the excluded patients is available. One patient in the RFCA was diagnosed with atrioventricular nodal re-entrant tachycardia during the procedure. At the discretion of the local investigator, he underwent successful slow pathway ablation, and no substrate-based ablation was performed. In the AAD group, 28 patients received amiodarone and 2 patients sotalol. One patient in the AAD group was lost from follow-up after 12 months due to relocation outside of the hospital district.

A total of 58 patients were randomly assigned to substrate-based RFCA (28 subjects) or AAD therapy (30 subjects) in 12 centres in four countries (*Figure 1*). Coronary artery disease and other underlying diseases were treated with guideline-directed medication. All patients were followed until death or for 24 months after starting the study treatment. The baseline characteristics of the patients were well balanced, and there were no statistically significant differences between those assigned to substrate-based RFCA vs. AAD therapy (*Table 1*). The mean age of the patients was 67 ± 7 years (range 47–78 years), and most of them were male (93%). The most common underlying diseases besides coronary artery disease were dyslipidaemia and hypertension. Left ventricular ejection fraction (LVEF) was 36 ± 11 (range 17–63%) and 37 ± 13 (range 17–61%), among those assigned to the RFCA and to the AAD therapy, respectively. The predominant locations of the prior MI were anterior (34%) and inferior wall (43%).

**Table 1:** Baseline characteristics of the patients

	Total (*n* = 58)	RFCA (*n* = 28)	AAD (*n* = 30)	*P*-value
Age (years)	66.6÷7.3	65.9 ± 8.2	67.1 ± 7.3	0.57
Men (*n*, %)	54 (93%)	26 (93%)	28 (93%)	1.00
Body mass index (kg/m^2^)	28.6 ± 4.0	28.8 ± 4.5	28.5 ± 3.6	0.79
LV ejection fraction (%)	37 ± 11	36 ± 11	37 ± 13	0.93
Time since first MI (years)	14.0 ± 8.9	13.4 ± 8.0	14.5 ± 8.9	0.19
Location of the MI				0.18
Anterior/septal	20 (34%)	13 (46%)	7 (23%)	
Inferior	25 (43%)	9 (32%)	16 (53%)	
Lateral	2 (3.4%)	0 (0%)	2 (6.7%)	
Posterior	3 (5.1%)	1 (21%)	2 (6.7%)	
Unknown	8 (14%)	5 (18%)	3 (10%)	
Diabetes	18 (31%)	9 (32%)	9 (30%)	0.89
Hypertension	36 (62%)	19 (68%)	17 (57%)	0.50
Dyslipidaemia	46 (79%)	24 (85%)	22 (73%)	0.34
Thromboembolic events^a^	9 (16%)	3 (11%)	6 (20%)	0.47
Atrial fibrillation/flutter	15 (26%)	6 (21%)	9 (30%)	0.78
Smoking				0.58
No	20 (34%)	11 (39%)	9 (30%)	
Former	24 (41%)	10 (36%)	14 (47%)	
Current	14 (24%)	7 (25%)	7 (23%)	
ICD indication				1.00
Primary prevention	20 (38%)	10 (36%)	10 (33%)	
Secondary prevention	36 (62%)	18 (60%)	20 (67%)	
ICD type				1.00
Single chamber	20 (34%)	9 (32%)	11 (37%)	
Dual chamber	31 (53%)	17 (61%)	15 (59%)	
CRT-D	7 (12%)	3 (11%)	4 (13%)	
Medication				
Beta-blocker	57 (98%)	28 (100%)	29 (97%)	
ACEI/ARB	53 (91%)	26 (93%)	27 (90%)	
Lipid-lowering medication	57 (98%)	28 (100%)	29 (97%)	
Digoxin	1 (0.8%)	0 (0%)	1 (3.3%)	0.74
Calcium channel blocker	9 (16%)	3 (11%)	7 (23%)	0.15
Antithrombotic medication^b^	56 (97%)	27 (96%)	29 (97%)	1.00
Laboratory and other measurements				
Systolic blood pressure	126 ± 19	122 ± 19	129 ± 18	0.14
Diastolic blood pressure	76 ± 11	77 ± 13	77 ± 9	0.46
eGFR^c^	79 ± 15	77 ± 15	80 ± 16	0.56
Creatine	92 ± 18	93 ± 12	92 ± 22	0.99
Sodium (mmol/L)	140 ± 2.8	140 ± 2.4	140 ± 3.1	0.76
Potassium (mmol/L)	4.2 ± 0.3	4.2 ± 0.2	4.3 ± 0.4	0.46
Pro-BNP	925 ± 1871	957 ± 1559	924 ± 1871	0.83
Troponin	48 ± 133	30 ± 35	63 ± 176	0.38

RFCA, radiofrequency catheter ablation; AAD, antiarrhythmic drug therapy; LV, left ventricular; MI, myocardial infarction; ICD, implantable cardioverter defibrillator; CRT-D, cardiac resynchronization therapy defibrillator; ACEI, angiotensin-converting enzyme inhibitor; ARB, angiotensin receptor blocker; OAC, oral anticoagulant; LMWH, low molecular weight heparin; eGFR, estimated glomerular filtration rate; BNP, brain natriuretic peptide.

^a^Stroke, transient ischaemic attack, and peripheral embolism.

^b^Aspirin, ATP receptor antagonist, oral anticoagulant of LMWH. cAccording to the CKD-EPI equation.

^c^According to the CKD-EPI equation.

### Therapeutic interventions

In the RFCA group, 27 patients underwent substrate-based RFCA. The procedure was performed using RMN in 19 patients (70%), and eight procedures (30%) were accomplished manually. One patient was diagnosed with a typical atrioventricular nodal re-entrant tachycardia (AVNRT). At the discretion of the local investigator, he underwent successful slow pathway ablation, and no substrate-based VT/VF ablation was performed. The mean duration of the substrate-based VT ablation procedure was 224 ± 60 min (range 130–332 min) and the mean fluoroscopy time 12.8 ± 8.2 min (range 3–29 min). Median length of hospitalization after the procedure was 1 day (range 0–5 days). No epicardial or right ventricular ablations were performed, and none of the patients underwent bipolar RF ablation or stereotactic body radiotherapy.

Abnormal fragmented signals and/or late potentials were identified in all patients who underwent 3D electroanatomic substrate mapping. The abnormal signals were successfully eliminated in 25 (93%) of the cases. In two patients, some fragmented signals and/or late potentials were not abolished despite extensive ablation. In these cases, ablation was stopped because no VT/VF was inducible. Overall, non-inducibility was reached in 20 of the 27 subjects (74%) who underwent substrate-based VT/VF ablation. A clinical VT was inducible in one patient whereas in six patients a non-clinical VT or VF was inducible.

In the AAD group, amiodarone was used in 28 patients (93%). There was no difference in the timing of the therapy initiation between the patients assigned to the catheter ablation compared to those treated with antiarrhythmic medication. The median amiodarone loading dose was 600 mg per day (range 100–1200 mg) and the maintenance dose after the 1–2 week loading period was 200 mg QD in all except for one subject (100 mg QD). Sotalol (120 mg BID) was used instead of amiodarone in two patients because of reduced pulmonary function at baseline testing, and in two patients, amiodarone was switched to sotalol due to due to amiodarone-induced hyperthyroidism during the follow-up.

In the AAD group, three patients (10%) crossed over to catheter ablation, and adjunctive antiarrhythmic drug therapy (sotalol) was used in two patients in the RFCA group (7.1%).

### Ventricular tachyarrhythmias

The median number of VT/VF episodes at 12 months (range 0–3 in the RFCA group vs. 0–22 in the AAD group) and at the extended 24 months follow-up (range 0–9 vs. 0–28 episodes) was zero in both groups (*P* = 0.45). Although the median VT/VF burden was zero in both groups, the distribution was highly skewed, and several patients experienced multiple VT/VF episodes. There were no significant associations between the baseline characteristics and the VT/VF burden.

**Figure 2:**
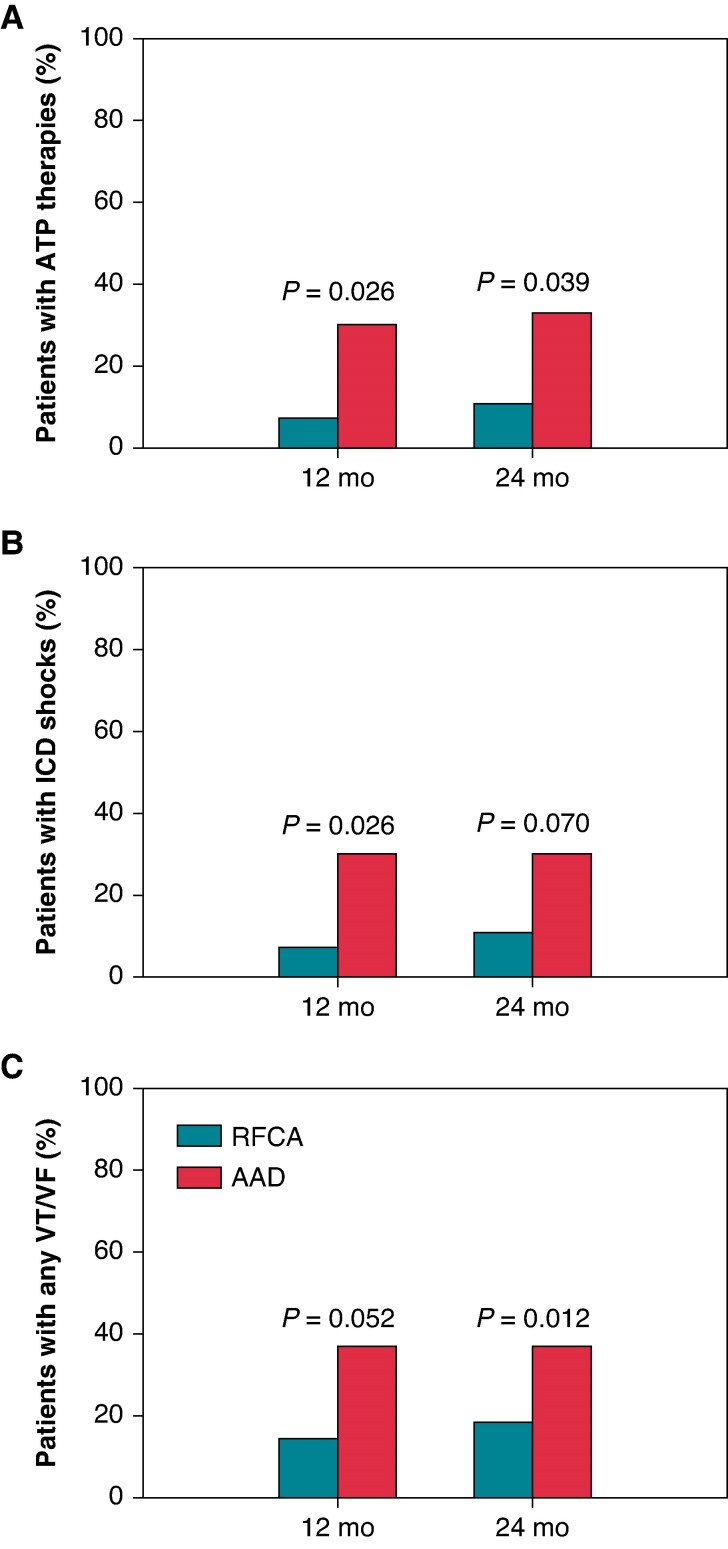
Patients with VT/VF during a 12- and 24-month follow-up. AAD, antiarrhythmic drug therapy; ATP, antitachycardia pacing; ICD, implantable cardioverter defibrillator; RFCA, radiofrequency catheter ablation.

At 12 months, ventricular tachyarrhythmias treated by ATP were reported in two patients in the RFCA and in nine patients in the AAD group (7% vs. 30%, *P* = 0.026), and at 24 months, ATP therapies had occurred in 3 and 11 patients in the RFCA and the AAD group (11% vs. 33%, *P* = 0.039), respectively (*Figure 2A*). The number of patients with adequate ICD shocks for VT/VF was lower in the RFCA (*n* = 2) than in the AAD (*n* = 9) group (7% vs. 30%, *P* = 0.026) at 12 months, but at 24 months, the difference was not statistically significant (11% vs. 30%, *P* = 0.07) (*Figure 2B*). During the extended follow-up, 23 patients (82%) in the RFCA group and 19 patients (63%) in the AAD group had no sustained VT/VF episodes (VT/VF burden zero) (*P* = 0.012) (*Figure 2C*). Ventricular tachyarrhythmias storm occurred in one patient in both groups.

### All cause and cardiovascular mortality and hospitalization

**Figure 3:**
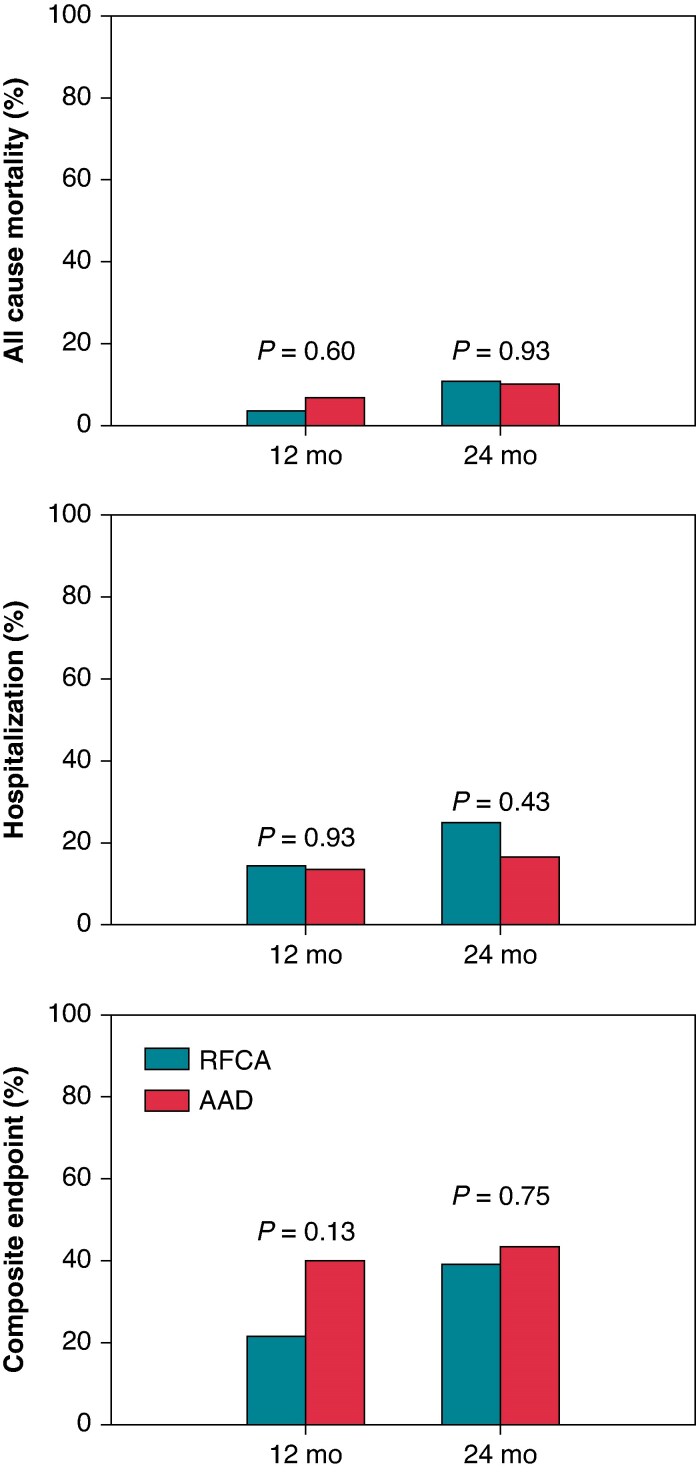
All-cause mortality, hospitalization, and a secondary composite endpoint of mortality, hospitalization, and VT/VF treated by the device during a 12- and 24-month follow-up.

There was no statistically significant difference in mortality between the groups (*Figure 3A*). At 24 months, death was reported in three patients (10.7%) in the RFCA group and in three patients (10.0%) in the AAD group (HR 1.02, 95% CI 0.20–5.11, *P* = 0.86). Time to death was 242 ± 154 days in the RFCA group and 521 ± 151 days in the AAD group (*P* = 0.14). Five deaths were caused by progressive hearth failure and one by acute intestinal ischaemia (see Supplementary material online, *Table S6*). In the RFCA group, acute reduction of left ventricular function was associated with fatal electric storm in one and in the AAD group in two patients.

At 12 months, four patients in both groups had been hospitalized at least once (14.2% in the RFCA group vs. 13.3% in the AAD group, *P* = 0.60). During the extended follow-up, seven patients (25%) in the RFCA group and five patients (17%) in the AAD group were hospitalized (HR 1.35, 95% CI 0.36–5.09. *P* = 0.66) (*Figure 3B*). The most common cause for hospitalization was ventricular arrhythmia in both groups, and the mean duration of hospitalization was 18 ± 21 days and 6 ± 6 days (*P* = 0.25) in the RFCA and the AAD group, respectively. In the RFCA group, one patient was hospitalized for 70 days due to severe subarachnoid haemorrhage. Time to first hospitalization was 259 ± 195 days in the RFCA and 196 ± 151 days in the AAD group (*P* = 0.57). No death or hospitalization was related to the assigned therapy.

The incidence of the secondary composite endpoint of VT/VF, all-cause mortality, and hospitalization was similar in the RFCA group (11 patients, 39%) and the AAD group (13 patients, 43%, *P* = 0.75) at 24 months (*Figure 3*).

### Adverse events

Non-fatal AEs were reported in 25% and 23% of the patients in the RFCA and the AAD group, respectively. The incidence of therapy-related AEs was 3.6% in the RFCA group (one at puncture site haematoma) and 16.7% in the AAD group (one patients with amiodarone-induced hypothyroidism, one with polyneuropathy and three with reduction in pulmonary function) (*P* = 0.10). The other AEs, which were not related to the assigned therapy, included progression of heart failure, shoulder pain, pneumonia, convulsions, and food poisoning.

## Discussion

The main finding of our study was that in antiarrhythmic drug-naïve patients with prior MI and a history of at least two VT/VFs episodes, the risk of ICD therapies was lower in the RFCA than in the AAD group. However, there was no meaningful difference in the primary outcome defined as the cumulative number of VT/VF episodes between the patients assigned to early substrate-based RFCA compared those treated with amiodarone. Likewise, there were no statistically significant differences between the groups in all-cause mortality and hospitalization.

### Ventricular tachyarrhythmias

About 10–50% of ICD recipients receive adequate ICD shocks within 3 years from the device implantation.^3,4^ It is evident that ICDs save lives, but the shocks are painful, and they have been associated with reduced quality of life, increase in mortality, and heart failure hospitalization.^26,27^ Given these findings and the growing number of ICD implantations, there is an urgent need to alleviate VT/VF burden in ICD recipients. Traditionally, antiarrhythmic drug therapy has been the mainstay for prevention of recurrent VT/VF episodes. However, due to inadequate efficacy and frequent adverse effects, drug therapy has been disappointing in the treatment of ventricular tachyarrhythmias.^28^

Lately, many investigators have evaluated the role of early catheter ablation in prevention of VT/VF recurrences among patients with ischaemic cardiomyopathy.^20,23,24,29^ According to the results of the VANISH-1 trial^21^ and recent meta-analyses,^30,31^ catheter ablation is superior to escalation of antiarrhythmic drug therapy in reducing appropriate ICD therapies among patients with ischaemic cardiomyopathy. The current study and the recently published VANISH-2 trial were designed to assess whether early substrate-based catheter ablation is more effective than antiarrhythmic medication as a first-line therapy for ventricular tachyarrhythmias in post-MI patients. In line with the VANISH-2 trial,^22^ we demonstrated that more patients in the RFCA than in the AAD group (82% vs. 63%) were free of adequate ICD therapies during the 2-year follow-up. These findings are clinically important although we found no meaningful difference in VT/VF burden between the groups. However, the distribution of VT/VF episodes was highly skewed, and interpretation of this endpoint in a small dataset should therefore be approached with caution. In particular, given the reported association between ICD discharges and poor outcomes,^26,27^ the finding that patients treated with RFCA were more often free of ICD shocks than those in the AAD group is imperative. Comparing our data to other VT ablation studies is demanding, because many of the earlier studies focused on prophylactic ablation in patients with no prior ICD therapies^12,32^ or included also patients already receiving antiarrhythmic medication.^30,31^

Unlike the VANISH trials^21,25^ and many other VT ablation studies, a good number of the catheter ablations in the current study were accomplished using RMN. The reported benefits of RMN over manual ablation techniques include reduced radiation exposure for both the patient and the operator and improved catheter manoeuvrability and stability in complex anatomy. In addition, RMN may reduce complication rates and procedural times without compromising efficacy. Comparison of the ablation techniques was beyond the scope of our study, but it is expected that in the near future, the results of the MAGNETIC VT trial^33^ will establish whether substrate-based VT ablation with RMN has clinical advantages over manual catheter manipulation.

### Mortality and hospitalization

In the current study, all-cause mortality at 24 months was about 10% with no meaningful difference between the treatment groups. However, like many earlier VT ablation trials, our study was not powered to detect statistically significant differences in mortality and hospitalization. In the recently published VANISH-2 trial,^25^ mortality rate at 2 years was about the same as in our study. The VANISH-2, which is the largest VT ablation trial, showed that an initial strategy of substrate-based RFCA led to lower risk of composite primary endpoint (i.e. death from any cause, appropriate ICD shock electric storm, or treated sustained VT below the detection limit of the ICD) than AAD therapy, but the difference in all-cause mortality remained statistically non-significant.

In our study, no perioperative deaths were reported. Nevertheless, prior studies have shown that risk of death is significantly higher in VT ablation than in atrial ablations, which should be taken into account when considering VT ablation.^34^

### Non-fatal adverse events

In the current study, non-fatal AEs were reported in about 25% of the patients with no meaningful difference between the groups. However, therapy-related AEs were slightly more common among those assigned to antiarrhythmic drug therapy. This difference might have been greater if the follow-up time had been longer, because the adverse effects related to catheter ablation commonly occur during or immediately after the procedure whereas the adverse effects of amiodarone increase with time. In keeping with prior data^7,35^ about 20% of the patients were not eligible to amiodarone or required discontinuation of the medication due to serious AEs attributed to the medication. In the RFCA group, only one procedure-related AE was reported (groin haematoma). Conversely, in a large German cohort, major AEs such as tamponade occurred after VT ablation in 5.3% of the patients, which is five-fold higher than in atrial fibrillation ablation.^34^

### Strengths and limitations of the study

An important strength of our study was that we included only antiarrhythmic drug naïve patients. On the other hand, our results are valid only for patient with remote MI and should not be extrapolated to those with acute MI or non-ischaemic cardiomyopathy. A major weakness of our study was small sample size. The power to detect statistically significant differences in VT/VF burden between the treatment groups was diminished, because like many other VT ablation trials,^36^ the current study was terminated prematurely due to slow recruitment. The reasons for slower than predicted enrolment varied between the centres. The COVID-19 pandemic was a major problem across all centres. In some centres, slow recruitment was related to competing research activities and in others the referring physicians preferred drug therapy over catheter ablation as the initial therapy for patients with only couple episodes of ventricular tachyarrhythmias. To overcome the problems caused by the smaller than anticipated study population, it was decided to wait until the extended follow-up was completed before reporting the results.

The techniques of VT ablation have evolved quickly after the protocol of the MANTRA-VT trial was designed. For example, multielectrode mapping catheters with small electrodes and short interelectrode distance facilitating identification of the ablation targets (i.e. fragmented signals and late potentials)^37^ were not widely available during the early phase of the current study, and they were used for mapping only in five patients. Likewise, recent evidence suggests that functional substrate identification such as decrement-evoked potential mapping may be able to identify the critical components of the VT circuit with a greater accuracy than conventional substrate mapping.^38,39^

On the other hand, although no new antiarrhythmic drugs for ventricular tachyarrhythmias have become available, medical therapy of heart failure has improved. Sodium-glucose cotransporter (SGLT2) inhibitors reduce mortality^40^ and potentially also the risk of serious ventricular tachyarrhythmias in patients with congestive heart failure.^41^ However, it is unlikely that these drugs would have had a group specific action in the current study. Finally, it has to be admitted that in a small study like this, VT/VF burden appeared to be a non-ideal primary endpoint as the distribution of the VT/VF episodes was highly skewed and many patients had no VT/VF episodes.

## Conclusions

Among patients with prior MI and few VT/VF recurrencies, early substrate-based RFCA reduced the risk of ICD therapies compared to the best available antiarrhythmic medication without compromising safety. This strengthens the role of catheter ablation as the initial treatment strategy for ventricular tachyarrhythmias in ischaemic cardiomyopathy.

## Supplementary Material

euaf236_Supplementary_Data

## Data Availability

The data underlying this article cannot be shared publicly, but it will be shared on reasonable request to the corresponding author.
