# Supplementary material for: Early substrate-based catheter ablation vs. antiarrhythmic drug therapy for ventricular tachyarrhythmias among patients with prior myocardial infarction: the MANTRA-VT randomized trial
Source: Europace. 2025 Oct 15;27(10):euaf236. doi: 10.1093/europace/euaf236 (PMC12527351; doi:10.1093/europace/euaf236)
Supplement: euaf236_Supplementary_Data [file euaf236_supplementary_data.pdf]

Supplementary materials

Table S1. Inclusion and exclusion criteria in the MANTRA-VT study.

**Inclusion criteria**	**Exclusion criteria**
• Age 18-80 years and prior myocardial infarction and ICD* for primary or secondary prevention of SCD<br>• At least two documented episodes of symptomatic VT/VF and the last one within the foregoing 12 months	• Age less than 18 years or more than 80 years<br>• Non-ischemic cardiomyopathy<br>• Ongoing chronic treatment with amiodarone<br>• Intolerance/contraindication to all class III antiarrhythmic drugs**<br>• Contraindication to endocardial catheter ablation<br>• Previous VT/VF ablation<br>• Open heart surgery within 3 months<br>• Prosthetic heart valve preventing access to left ventricle<br>• Planned revascularization (PCI or CABG), surgery for structural heart disease or heart transplantation<br>• Pregnancy or planned pregnancy within the follow-up period<br>• Secondary cause for VT/VF (e.g., acute myocardial infarction)<br>• Patient does not want to participate<br>• Life expectancy less than 12 months

*ICD (implantable cardioverter defibrillator): single chamber (VVI), dual chamber (DDD) or ICD with biventricular pacing capability (CRT-D)

**If amiodarone is contraindicated sotalol can be used.

CABG = coronary artery bypass grafting; PCI = percutaneous coronary intervention; SCD = sudden cardiac death; VT = ventricular tachycardia; VF = ventricular fibrillation

The location and size of the prior MI and underlying rhythm (*e.g.*, sinus rhythm or atrial fibrillation) did not affect inclusion.

Table S2. Patient follow-up schedule in the MANTRA-VT study.

	Clinical visit							
**Investigation**	**Screening**	**Baseline**	**Visit 1**	**Visit 2**	**Visit 3**	**Visit 4**	**Visit 5**	**Visit 7**
			Discharge	3 mo ± 2 wk	6 mo ± 2 wk	12 mo ± 2 wk	18 mo ± 2 wk	24 mo ± 2 wk
Informed consent	x							
Inclusion/Exclusion criteria	x							
Randomization	x							
Medical history		x						
Clinical events		x	x	x	(x)	x	(x)	x
Patient event report			(x)	x	(x)	x	(x)	x
Concomitant medication		x	x	x	x	x	x	x
Physical examination		x	x	x	(x)	x	(x)	x
12 lead ECG		x	x	x	(x)	x	(x)	x
Laboratory tests		x	(x)	x		x		x
Echocardiography		x	x	x			x	x
ICD interrogation		x	x	x	(x)	x	(x)	x
24-hour Holter		(x)				(x)		
Quality of life								
SF-36		x				x		x

EQ-5D		x				x		x
Patient related outcomes								
Depression (PHQ-9)		x				x		x
Anxiety (GAD-7)		X				x		x
ICDC-8		x				x		X
Expect-ICD		X				x		x
Type D personality (DS14)		x				x		x

Laboratory tests included Hb, leucocytes, thrombocytes, liver tests, thyroid tests, NT pro-BNP and CRP. Clinical visits at 6 and 18 months could be replaced by phone contact and remote ICD evaluation. More details on the quality of life and other patient related outcome questionnaires are presented in the text. Abbreviations: ICD = implantable cardioverter defibrillator.

Table S3. Prespecified secondary endpoints in the MANTRA-VT trial.

All-cause mortality
Cardiovascular mortality
Hospitalization (cause, time to first hospitalization, length of hospitalizations)
Ablation/antiarrhythmic medication related side effects including proarrhythmic events
Health economics (including planned and unplanned hospitalization and out patients visits, (type, length and number of antiarrhythmic drugs)
Quality of life (SF-36, EQ 5D)
Patient related outcome
Number of appropriate ICD therapies and documented sustained VT or VF episodes at 24 months
Number of non-sustained VTs detected by the device
Incidence of electric storm
Time to first VT/VF episode
Time to reablation or change of antiarrhythmic medication (*i.e.*, crossover)
Efficacy of ICD therapies
Inappropriate ICD therapies
Incidence of atrial fibrillation and other sustained supraventricular tachyarrhythmias

Table S4. Classification of the adverse events (AE) in the MANTRA-VT study.

A.	Serious Adverse Event	Any clinical event resulting in death, a life-threatening complication, or a persistent or significant disability/incapacity that requires hospitalization, prolongs hospitalization or requires intervention to prevent a permanent impairment of a body function or damage to a body structure.
B.	Non-Serious Adverse Event	All AE's not meeting the definition of a serious AE, including events resulting in a transient impairment of a body function that resolves with minimal intervention. Non-serious AE's must be reported to the coordinating investigator (PR) using the AE CRF.
C.	Known Adverse Event	An event that has been reported in previous studies of RFA or AAD treatment. Adverse events that are known for RFA include the following<br>• Local hematoma or ecchymosis<br>• Arteriovenous fistula<br>• Pseudoaneurysm<br>• Thromboembolism<br>• Vasovagal reaction<br>• Pneumothorax<br>• Haemothorax<br>• Infection<br>• Endocarditis

	• Pulmonary vein stenosis/occlusion<br>• Chest pain or discomfort<br>• Complete heart block<br>• Ventricular pro-arrhythmia<br>• Pulmonary or systemic thromboembolism<br>• Air embolism<br>• Arrhythmias<br>• Valvular damage or insufficiency<br>• Pericardial effusion<br>• Transient ischemic attack<br>• Cerebrovascular accident<br>• Cardiac perforation or tamponade<br>• Pericarditis<br>Death
D. Unanticipated Adverse Event	An event not previously identified in nature, severity or degree of incidence in this protocol. Any other serious problem associated with the treatment that relates to the rights, safety or welfare of subjects.

1

Table S5. Power calculations for various options including 2±2, 3±2 and 4±2 therapies in the MANTRA-VT study. The reduction of the VT/VF episodes by catheter ablation was expected to be 35-50% and the calculations were made for both the high and low estimate. According to the most like scenario (2±2 episodes and 50 % reduction) 51 patients per group should be enough to show statistically significant difference between the RFCA and the AAD therapy. n=number of patients per group

Reduction	Power		n for 2±2 Tx	n for 3±2 Tx	n for 4±2 Tx
25%	80%	One-sided	199	89	51
25%	80%	Two-sided	253	113	64
25%	90%	One-sided	275	123	70
25%	90%	Two-sided	338	151	86
50%	80%	One-sided	51	23	14
50%	80%	Two-sided	64	29	17
50%	90%	One-sided	70	32	18
50%	90%	Two-sided	86	39	23

Table S5. Cause of death and hospitalisation.

Study group	Cause of death	Cause of hospitalisation
RFCA	Progressive heart failure (n=3	Ventricular tachyarrhythmia (n=4)<br>Subarachnoid haemorrhage (n=1)<br>Pneumonia (n=1)<br>NSTEMI (n=1)
AAD	Progressive heart failure (n=2)<br>Intestinal ischaemia (n=1)	Ventricular arrhythmia (n=2)<br>ICD upgrade to CRT-D (n=1)<br>Congestive heart failure (n=1<br>Programmed electrical stimulation to evaluate the mechanism of atrial tachyarrhythmia (n=1)

RFCA = radiofrequency catheter ablation; AAD = antiarrhythmic drug therapy; NSTEMI = non-ST elevation myocardial infarction; ICD = implantable cardioverter defibrillator; CRT-D = cardiac resynchronisation therapy defibrillator.

Table S6. Assessment of the outcome and causality of the adverse events (AE).

Outcome	• Resolved: patient fully recovered with no observable residual effects<br>• Improved: patient’s condition improved but residual effects remain<br>• Unchanged: AE is ongoing<br>• Worsened: patient’s overall condition worsened<br>• Hospitalization: AE required or prolonged hospitalization<br>• Permanent disability: AE resulted in a permanent impairment of a body function or damage to a body structure.
Causality	• Therapy-related (RF ablation or antiarrhythmic medication): the event is directly related by timing and/or pathophysiology with the therapeutic procedures described in this protocol<br>• Possibly therapy-related: the AE may be associated with the therapeutic procedures described in this protocol by timing and/or pathophysiology<br>• Not related: the AE is not associated with the therapeutic procedures described in this protocol.
